# [1,2-Bis(diphenyl­phosphino)-1,2-dicarba-*closo*-dodeca­borane-κ^2^ *P*,*P*′][7,8-bis­(di­phenyl­phosphino)-7,8-dicarba-*nido*-undeca­borato-κ^2^ *P*,*P*′]gold(I)–dichloro­methane–water (2/1/1)

**DOI:** 10.1107/S1600536809014937

**Published:** 2009-04-30

**Authors:** Joseph A. Ioppolo, Jack K. Clegg, Louis M. Rendina

**Affiliations:** a School of Chemistry, F11, The University of Sydney, New South Wales 2006, Australia; b Centre for Heavy Metals Research, School of Chemistry, F11, The University of Sydney, New South Wales 2006, Australia

## Abstract

The title compound, [Au(C_26_H_30_B_10_P_2_)(C_26_H_30_B_9_P_2_)]·0.5CH_2_Cl_2_·0.5H_2_O, contains two independent complex mol­ecules in the asymmetric unit. The gold(I) centres display a distorted tetra­hedral geometry. The complex is stablized through weak intra­molecular π–π stacking (*Cg*⋯*Cg* = 4.17 Å) and edge-to-face inter­actions (H⋯*Cg* = 3.21 Å). Adjacent mol­ecules inter­act through C—H⋯π (H⋯*Cg* = 2.88 Å) and B—H⋯π (H⋯*Cg* = 3.15 Å) contacts, forming a three-dimensional network, with solvent mol­ecules occupying the cavities. One of the phenyl groups was disordered over two sites with occupancy factors of 0.65 and 0.35.

## Related literature

The chelating P-donor ligand 1,2-bis­(diphenyl­phosphino)-1,2-dicarba-*closo*-carborane has been used to prepare 2-, 3- and 4-coordinate complexes of gold(I) (Crespo *et al.*, 1992 ▶, 1994 ▶; Al-Baker *et al.*, 1985 ▶). Coordination of this ligand has often led to deboronation of the *closo*-carborane cage to afford the corresponding *nido*-carborane in polar solvents (Teixidor *et al.*, 1995 ▶, 1996 ▶). A non-solvated crystal structure of the title compound has been reported previously (Crespo *et al.*, 1997 ▶). Facile deboronation of the carborane cage in polar solvents has also been observed when substituents α to the cage are electron withdrawing (Shaeck & Kahl, 1999 ▶; Ioppolo *et al.*, 2007*a* ▶,*b* ▶). In contrast, our group has shown that ligands containing the thermodynamically stable 1,12-carborane cluster do not degrade upon complexation to gold(I) (Ioppolo *et al.*, 2007*a* ▶,*b* ▶). Boron-containing ligands and their respective complexes are of inter­est for potential application in boron neutron capture therapy (BNCT) (Crossley *et al.*, 2005 ▶, 2007 ▶; Todd *et al.*, 2005 ▶; Ioppolo *et al.*, 2007*a* ▶,*b* ▶; Ching *et al.*, 2007 ▶). For the synthesis of the precursor gold compound, see: Uson *et al.* (1989 ▶).
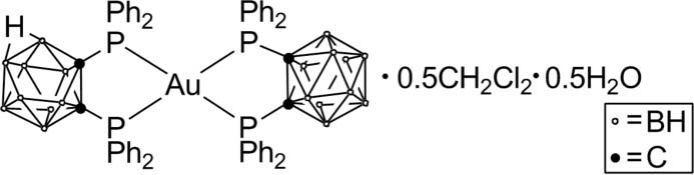


## Experimental

### 

#### Crystal data

[Au(C_26_H_30_B_10_P_2_)(C_26_H_30_B_9_P_2_)]·0.5CH_2_Cl_2_·0.5H_2_O
*M* *_r_* = 1262.71
Triclinic, 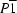

*a* = 13.2043 (6) Å
*b* = 20.1424 (9) Å
*c* = 23.5721 (11) Å
α = 102.131 (3)°
β = 90.849 (3)°
γ = 105.839 (2)°
*V* = 5879.3 (5) Å^3^
*Z* = 4
Mo *K*α radiation
μ = 2.69 mm^−1^
*T* = 150 K
0.10 × 0.10 × 0.02 mm

#### Data collection

Bruker APEXII diffractometer
Absorption correction: multi-scan (*SADABS*; Sheldrick, 2003 ▶) *T*_min_ = 0.767, *T*_max_ = 0.948
157624 measured reflections
20684 independent reflections
14971 reflections with *I* > 2σ(*I*)
*R*_int_ = 0.106

#### Refinement

*R*[*F* ^2^ > 2σ(*F* ^2^)] = 0.044
*wR*(*F* ^2^) = 0.094
*S* = 1.06
20684 reflections
1397 parameters
77 restraints
H atoms treated by a mixture of independent and constrained refinement
Δρ_max_ = 1.47 e Å^−3^
Δρ_min_ = −1.00 e Å^−3^

### 

Data collection: *APEX* (Bruker, 2003 ▶); cell refinement: *SAINT* (Bruker, 2003 ▶); data reduction: *SAINT* and *XPREP* (Bruker, 2003 ▶); program(s) used to solve structure: *SIR97* (Altomare *et al.*, 1999 ▶); program(s) used to refine structure: *SHELXL97* (Sheldrick, 2008 ▶); molecular graphics: *ORTEP-3* (Farrugia, 1997 ▶), *WinGX32* (Farrugia, 1999 ▶), *POV-RAY* (Cason, 2002 ▶) and *WebLab ViewerPro* (Molecular Simulations, 2000 ▶); software used to prepare material for publication: *enCIFer* (Allen *et al.*, 2004 ▶).

## Supplementary Material

Crystal structure: contains datablocks global, I. DOI: 10.1107/S1600536809014937/tk2431sup1.cif

Structure factors: contains datablocks I. DOI: 10.1107/S1600536809014937/tk2431Isup2.hkl

Additional supplementary materials:  crystallographic information; 3D view; checkCIF report

## Figures and Tables

**Table 1:** Selected bond angles (°)

P3—Au1—P2	119.51 (5)
P3—Au1—P1	131.67 (5)
P2—Au1—P1	86.45 (5)
P3—Au1—P4	89.19 (5)
P2—Au1—P4	131.50 (5)
P1—Au1—P4	103.15 (5)
P6—Au2—P7	119.47 (5)
P6—Au2—P8	131.52 (5)
P7—Au2—P8	88.03 (5)
P6—Au2—P5	84.82 (5)
P7—Au2—P5	136.50 (5)
P8—Au2—P5	102.13 (6)

**Fig. 1.**
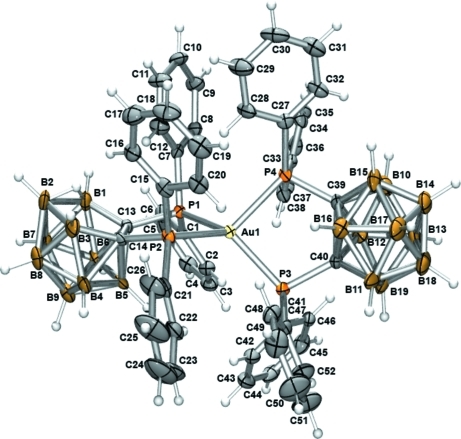
ORTEP representation of one of the two crystallographically independent molecules in the asymmetric unit of the structure of (I) shown with 50% probability ellipsoids. Solvent molecules are omitted for clarity.

**Fig. 2.**
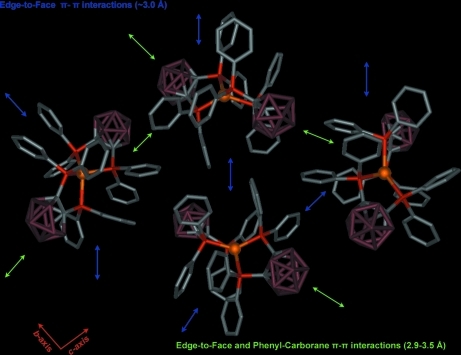
A schematic representation of part of two-dimensional sheets formed by π-π interactions.

**Fig. 3.**
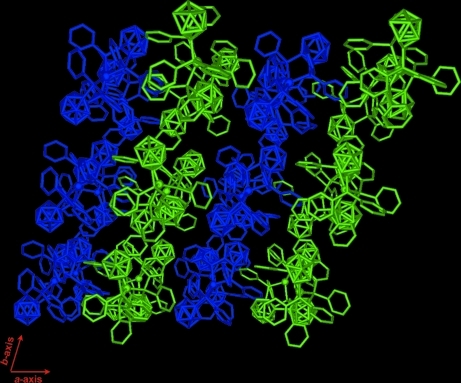
A schematic representation of the extended crystal packing. Adjacent layers (as shown in Fig 2) are given in alternating colours.

## supplementary crystallographic information

### Comment

We are currently investigating a wide variety of boron-containing ligands (Crossley *et al.*, 2005; Todd *et al.*, 2005; Ioppolo *et al.*, 2007a, 2007b; Ching *et al.*, 2007) for application as potential BNCT agents (Crossley *et al.*, 2007). The title compound (I) was synthesized as part of these ongoing investigations. The gold(I) centre is bound to four phosphorus atoms from two ligands (one 1,2-bis(diphenylphosphino)-1,2-dicarba-*closo*-dodecaborane and one 7,8-bis(diphenylphosphino)-7,8-dicarba-*nido*-undecaborane) giving an overall neutral charge to each molecule. The metal centre has a distorted tetrahedral geometry (Fig. 1), the distortion arising primarily from the small bite angle of the phosphorus containing ligands of less than 90 °. This arrangement is stabilized by intramolecular edge-to-face and offset face-to-face π-π interactions between the phenyl rings bound to each phosphorus ligand. There are further intermolecular π-π interactions present in the crystal lattice. Adjacent molecules connect *via* both edge-to-face π-π and phenyl-carborane interactions forming infinite two-dimensional sheets which propagate in the *bc*-plane. Indicative distances include C70H—B13 (2.86 Å), C102H—B38 (3.28 Å), C30H—C88 (2.88 Å) and C18H—C63 (3.17 Å). A schematic representation of part of one of these sheets is given in Fig. 2. These 2-D sheets interact with adjacent sheets through further BH - π and phenyl-phenyl contacts to form a 3-D motif, a schematic representation of which is shown in Fig. 3. These interactions are indicated by a B35H—C100 distance of 3.15 Å and a C24H—C70 distance of 3.53 Å.

### Experimental

The title compound was prepared from [AuCl(SMe_2_)] by using a modification of a previously reported method (Uson *et al.*, 1989) and identified as the desired product by comparison with literature data (Crespo *et al.*, 1997). Crystals suitable for X-ray diffraction were isolated from a CH_2_Cl_2_ solution after several days of slow evaporation.

### Refinement

C and B bound-H (except the bridging H present on the *nido* cages) atoms were included in idealized positions and refined using a riding-model approximation, with C—H = 0.95 - 0.99 Å and B—H = 1.12 Å. The bridging H atoms on the *nido* cages were located in the difference Fourier map prior and refined with bond length restraints of 1.10 (4) Å. *U*_iso_(H) values were fixed at 1.2*U*_eq_ of the parent atoms. One of the phenyl rings is disordered and modelled over two positions with occupancies of 0.65 and 0.35, respectively. Rigid body restraints were employed on these rings to facilitate realistic modelling. The two water molecules are both half occupancy and were modelled isotropically. Despite being in almost ideal positions for hydrogen bonding, their H atoms could not be located in the difference Fourier map, and were not included in the model. The max. and min. electron density peaks were located 0.91 Å and 0.07 Å from the Au2 and C56B atoms, respectively.

### Crystal data

**Table:**

[Au(C_26_H_30_B_10_P_2_)(C_26_H_30_B_9_P_2_)]·0.5CH_2_Cl_2_·0.5H_2_O	*Z* = 4
*M**_r_* = 1262.71	*F*(000) = 2528
Triclinic, *P*1	*D*_x_ = 1.427 Mg m^−^^3^
Hall symbol: -P 1	Mo *K*α radiation, λ = 0.71073 Å
*a* = 13.2043 (6) Å	Cell parameters from 5169 reflections
*b* = 20.1424 (9) Å	θ = 3.0–22.5°
*c* = 23.5721 (11) Å	µ = 2.69 mm^−^^1^
α = 102.131 (3)°	*T* = 150 K
β = 90.849 (3)°	Plate, colourless
γ = 105.839 (2)°	0.10 × 0.10 × 0.02 mm
*V* = 5879.3 (5) Å^3^	

### Data collection

**Table:**

Bruker APEXII FR591 diffractometer	20684 independent reflections
Radiation source: rotating anode	14971 reflections with *I* > 2σ(*I*)
graphite	*R*_int_ = 0.106
ω and φ scans	θ_max_ = 25.0°, θ_min_ = 1.1°
Absorption correction: multi-scan (*SADABS*; Sheldrick, 2003)	*h* = −15→15
*T*_min_ = 0.767, *T*_max_ = 0.948	*k* = −23→23
157624 measured reflections	*l* = −28→28

### Refinement

**Table:**

Refinement on *F*^2^	Primary atom site location: structure-invariant direct methods
Least-squares matrix: full	Secondary atom site location: difference Fourier map
*R*[*F*^2^ > 2σ(*F*^2^)] = 0.044	Hydrogen site location: inferred from neighbouring sites
*wR*(*F*^2^) = 0.094	H atoms treated by a mixture of independent and constrained refinement
*S* = 1.06	*w* = 1/[σ^2^(*F*_o_^2^) + (0.0344*P*)^2^ + 11.0634*P*] where *P* = (*F*_o_^2^ + 2*F*_c_^2^)/3
20684 reflections	(Δ/σ)_max_ = 0.001
1397 parameters	Δρ_max_ = 1.47 e Å^−^^3^
77 restraints	Δρ_min_ = −1.00 e Å^−^^3^

### Special details

**Table:**

Experimental. The crystal was coated in Exxon Paratone N hydrocarbon oil and mounted on a thin mohair fibre attached to a copper pin. Upon mounting on the diffractometer, the crystal was quenched to 150(K) under a cold nitrogen gas stream supplied by an Oxford Cryosystems Cryostream and data were collected at this temperature.
Geometry. All e.s.d.'s (except the e.s.d. in the dihedral angle between two l.s. planes) are estimated using the full covariance matrix. The cell e.s.d.'s are taken into account individually in the estimation of e.s.d.'s in distances, angles and torsion angles; correlations between e.s.d.'s in cell parameters are only used when they are defined by crystal symmetry. An approximate (isotropic) treatment of cell e.s.d.'s is used for estimating e.s.d.'s involving l.s. planes.
Refinement. Refinement of *F*^2^ against ALL reflections. The weighted *R*-factor *wR* and goodness of fit *S* are based on *F*^2^, conventional *R*-factors *R* are based on *F*, with *F* set to zero for negative *F*^2^. The threshold expression of *F*^2^ > σ(*F*^2^) is used only for calculating *R*-factors(gt) *etc*. and is not relevant to the choice of reflections for refinement. *R*-factors based on *F*^2^ are statistically about twice as large as those based on *F*, and *R*- factors based on ALL data will be even larger.

### Fractional atomic coordinates and isotropic or equivalent isotropic displacement parameters (Å^2^)

**Table:**

	*x*	*y*	*z*	*U*_iso_*/*U*_eq_	Occ. (<1)
C1	0.1097 (4)	0.8335 (3)	0.0588 (2)	0.0191 (12)	
C2	0.1375 (4)	0.9003 (3)	0.0470 (3)	0.0256 (13)	
H2	0.1433	0.9053	0.0079	0.031*	
C3	0.1570 (5)	0.9601 (3)	0.0921 (3)	0.0323 (15)	
H3	0.1770	1.0058	0.0838	0.039*	
C4	0.1477 (5)	0.9533 (3)	0.1488 (3)	0.0336 (15)	
H4	0.1606	0.9944	0.1794	0.040*	
C5	0.1197 (4)	0.8874 (4)	0.1612 (3)	0.0329 (16)	
H5	0.1129	0.8829	0.2004	0.039*	
C6	0.1014 (4)	0.8278 (3)	0.1169 (3)	0.0280 (14)	
H6	0.0829	0.7824	0.1259	0.034*	
C7	0.1386 (4)	0.6922 (3)	0.0252 (2)	0.0221 (13)	
C8	0.2220 (4)	0.7112 (3)	0.0671 (3)	0.0276 (14)	
H8	0.2504	0.7596	0.0862	0.033*	
C9	0.2647 (5)	0.6608 (3)	0.0816 (3)	0.0354 (16)	
H9	0.3214	0.6746	0.1109	0.042*	
C10	0.2253 (5)	0.5905 (3)	0.0538 (3)	0.0378 (17)	
H10	0.2557	0.5561	0.0631	0.045*	
C11	0.1404 (5)	0.5701 (3)	0.0117 (3)	0.0397 (17)	
H11	0.1121	0.5219	−0.0076	0.048*	
C12	0.0984 (5)	0.6210 (3)	−0.0013 (3)	0.0320 (15)	
H12	0.0399	0.6069	−0.0293	0.038*	
C13	−0.0583 (4)	0.7145 (3)	−0.0081 (2)	0.0185 (12)	
C14	−0.1161 (4)	0.6758 (3)	−0.0725 (2)	0.0216 (13)	
C15	−0.0209 (4)	0.5873 (3)	−0.1640 (3)	0.0282 (14)	
C16	−0.0579 (4)	0.5281 (3)	−0.1396 (3)	0.0306 (15)	
H16	−0.0930	0.5322	−0.1047	0.037*	
C17	−0.0428 (6)	0.4637 (4)	−0.1666 (3)	0.0467 (19)	
H17	−0.0686	0.4238	−0.1500	0.056*	
C18	0.0081 (6)	0.4563 (4)	−0.2166 (3)	0.0469 (19)	
H18	0.0170	0.4117	−0.2347	0.056*	
C19	0.0467 (5)	0.5149 (4)	−0.2403 (3)	0.0431 (17)	
H19	0.0829	0.5104	−0.2748	0.052*	
C20	0.0329 (5)	0.5794 (3)	−0.2144 (3)	0.0321 (15)	
H20	0.0604	0.6191	−0.2309	0.039*	
C21	−0.1060 (4)	0.6944 (3)	−0.1915 (3)	0.0289 (14)	
C22	−0.1117 (5)	0.7626 (4)	−0.1883 (3)	0.0395 (17)	
H22	−0.0756	0.7986	−0.1560	0.047*	
C23	−0.1670 (6)	0.7807 (5)	−0.2296 (4)	0.057 (2)	
H23	−0.1705	0.8279	−0.2253	0.068*	
C24	−0.2166 (7)	0.7297 (6)	−0.2766 (4)	0.081 (3)	
H24	−0.2562	0.7415	−0.3051	0.097*	
C25	−0.2105 (7)	0.6613 (6)	−0.2838 (4)	0.079 (3)	
H25	−0.2415	0.6268	−0.3181	0.095*	
C26	−0.1577 (5)	0.6431 (4)	−0.2399 (3)	0.0490 (19)	
H26	−0.1573	0.5954	−0.2432	0.059*	
C27	0.3423 (4)	0.6619 (3)	−0.1153 (2)	0.0239 (13)	
C28	0.2528 (5)	0.6040 (3)	−0.1205 (3)	0.0334 (15)	
H28	0.1848	0.6113	−0.1175	0.040*	
C29	0.2634 (5)	0.5360 (4)	−0.1300 (3)	0.050 (2)	
H29	0.2027	0.4968	−0.1325	0.060*	
C30	0.3622 (6)	0.5248 (4)	−0.1358 (3)	0.0465 (19)	
H30	0.3693	0.4782	−0.1428	0.056*	
C31	0.4507 (5)	0.5827 (4)	−0.1315 (3)	0.0412 (17)	
H31	0.5184	0.5753	−0.1363	0.049*	
C32	0.4412 (5)	0.6503 (3)	−0.1202 (3)	0.0298 (14)	
H32	0.5026	0.6895	−0.1158	0.036*	
C33	0.3867 (4)	0.7909 (3)	−0.0243 (2)	0.0210 (13)	
C34	0.4573 (4)	0.7648 (3)	0.0028 (3)	0.0274 (14)	
H34	0.4729	0.7228	−0.0168	0.033*	
C35	0.5044 (5)	0.7989 (3)	0.0574 (3)	0.0299 (14)	
H35	0.5535	0.7810	0.0750	0.036*	
C36	0.4808 (4)	0.8594 (3)	0.0871 (3)	0.0296 (14)	
H36	0.5139	0.8830	0.1248	0.035*	
C37	0.4100 (4)	0.8848 (3)	0.0620 (3)	0.0311 (15)	
H37	0.3929	0.9257	0.0826	0.037*	
C38	0.3631 (4)	0.8512 (3)	0.0068 (3)	0.0272 (14)	
H38	0.3140	0.8695	−0.0103	0.033*	
C39	0.3949 (4)	0.8013 (3)	−0.1451 (3)	0.0245 (13)	
C40	0.3374 (4)	0.8589 (3)	−0.1681 (3)	0.0257 (14)	
C41	0.2055 (4)	0.9455 (3)	−0.0994 (2)	0.0199 (12)	
C42	0.1073 (5)	0.9581 (3)	−0.0936 (3)	0.0319 (15)	
H42	0.0470	0.9255	−0.1161	0.038*	
C43	0.0970 (5)	1.0174 (4)	−0.0555 (3)	0.0415 (18)	
H43	0.0297	1.0260	−0.0528	0.050*	
C44	0.1825 (5)	1.0645 (3)	−0.0212 (3)	0.0392 (17)	
H44	0.1742	1.1044	0.0061	0.047*	
C45	0.2798 (5)	1.0529 (3)	−0.0268 (3)	0.0372 (16)	
H45	0.3396	1.0860	−0.0041	0.045*	
C46	0.2922 (4)	0.9938 (3)	−0.0651 (3)	0.0284 (14)	
H46	0.3600	0.9861	−0.0680	0.034*	
C47	0.1366 (4)	0.8565 (3)	−0.2141 (3)	0.0288 (14)	
C48	0.1377 (6)	0.9187 (4)	−0.2319 (3)	0.0448 (18)	
H48	0.1677	0.9634	−0.2063	0.054*	
C49	0.0949 (7)	0.9147 (5)	−0.2867 (3)	0.062 (2)	
H49	0.0935	0.9569	−0.2983	0.074*	
C50	0.0543 (7)	0.8503 (5)	−0.3248 (3)	0.066 (2)	
H50	0.0275	0.8481	−0.3629	0.080*	
C51	0.0526 (6)	0.7886 (4)	−0.3074 (3)	0.051 (2)	
H51	0.0233	0.7440	−0.3333	0.061*	
C52	0.0933 (5)	0.7918 (3)	−0.2527 (3)	0.0340 (15)	
H52	0.0919	0.7492	−0.2410	0.041*	
C58A	0.3300 (5)	0.4932 (5)	0.6977 (4)	0.0417 (13)	0.65
H58A	0.2662	0.4764	0.7150	0.050*	0.65
C53A	0.3750 (7)	0.4455 (3)	0.6634 (4)	0.0417 (13)	0.65
C55A	0.4684 (6)	0.4701 (3)	0.6381 (3)	0.0417 (13)	0.65
H55A	0.4992	0.4375	0.6146	0.050*	0.65
C56A	0.5167 (5)	0.5424 (3)	0.6472 (3)	0.0417 (13)	0.65
H56A	0.5805	0.5592	0.6300	0.050*	0.65
C57A	0.4717 (5)	0.5901 (3)	0.6816 (3)	0.0417 (13)	0.65
H57A	0.5047	0.6396	0.6878	0.050*	0.65
C54A	0.3783 (6)	0.5655 (4)	0.7068 (4)	0.0417 (13)	0.65
H54A	0.3475	0.5981	0.7303	0.050*	0.65
C53B	0.3764 (13)	0.4424 (8)	0.6679 (11)	0.080 (4)	0.35
C58B	0.3352 (10)	0.4964 (11)	0.6951 (12)	0.080 (4)	0.35
H58B	0.2612	0.4874	0.6980	0.096*	0.35
C55B	0.4022 (13)	0.5636 (10)	0.7181 (10)	0.080 (4)	0.35
H55B	0.3740	0.6005	0.7366	0.096*	0.35
C54B	0.5105 (12)	0.5768 (7)	0.7138 (8)	0.080 (4)	0.35
H54B	0.5562	0.6227	0.7295	0.096*	0.35
C56B	0.5517 (10)	0.5228 (8)	0.6866 (9)	0.080 (4)	0.35
H56B	0.6257	0.5318	0.6837	0.096*	0.35
C57B	0.4847 (14)	0.4556 (7)	0.6637 (9)	0.080 (4)	0.35
H57B	0.5129	0.4187	0.6451	0.096*	0.35
C59	0.2188 (4)	0.3317 (3)	0.7009 (3)	0.0281 (14)	
C60	0.1181 (6)	0.2851 (4)	0.6858 (3)	0.0483 (19)	
H60	0.0930	0.2672	0.6460	0.058*	
C61	0.0541 (6)	0.2648 (4)	0.7297 (4)	0.058 (2)	
H61	−0.0147	0.2330	0.7197	0.070*	
C62	0.0915 (5)	0.2912 (4)	0.7878 (3)	0.0427 (18)	
H62	0.0504	0.2753	0.8176	0.051*	
C63	0.1865 (5)	0.3395 (4)	0.8014 (3)	0.0436 (18)	
H63	0.2104	0.3599	0.8411	0.052*	
C64	0.2500 (5)	0.3597 (3)	0.7580 (3)	0.0376 (16)	
H64	0.3167	0.3939	0.7685	0.045*	
C65	0.2140 (5)	0.3413 (3)	0.5808 (3)	0.0317 (15)	
C66	0.1840 (5)	0.2670 (3)	0.5331 (2)	0.0303 (15)	
C67	0.2875 (5)	0.1684 (3)	0.4721 (3)	0.0306 (15)	
C68	0.3782 (5)	0.2068 (4)	0.4515 (3)	0.0429 (18)	
H68	0.4260	0.2461	0.4770	0.051*	
C69	0.3987 (7)	0.1879 (5)	0.3941 (4)	0.062 (2)	
H69	0.4579	0.2159	0.3792	0.074*	
C70	0.3321 (8)	0.1274 (5)	0.3581 (4)	0.073 (3)	
H70	0.3468	0.1139	0.3187	0.088*	
C71	0.2467 (7)	0.0876 (4)	0.3785 (3)	0.059 (2)	
H71	0.2024	0.0460	0.3537	0.071*	
C72	0.2242 (6)	0.1079 (4)	0.4356 (3)	0.0442 (18)	
H72	0.1644	0.0798	0.4499	0.053*	
C73	0.1599 (4)	0.1240 (3)	0.5615 (2)	0.0300 (15)	
C74	0.0556 (5)	0.1196 (4)	0.5766 (3)	0.0380 (16)	
H74	0.0268	0.1578	0.5761	0.046*	
C75	−0.0054 (5)	0.0580 (4)	0.5924 (3)	0.049 (2)	
H75	−0.0761	0.0544	0.6018	0.059*	
C76	0.0369 (6)	0.0029 (4)	0.5943 (3)	0.057 (2)	
H76	−0.0042	−0.0381	0.6058	0.069*	
C77	0.1390 (6)	0.0071 (4)	0.5795 (4)	0.061 (2)	
H77	0.1676	−0.0311	0.5804	0.073*	
C78	0.1997 (5)	0.0666 (4)	0.5633 (3)	0.0441 (18)	
H78	0.2697	0.0688	0.5531	0.053*	
C79	0.3188 (4)	0.1737 (3)	0.7488 (3)	0.0297 (14)	
C80	0.2186 (5)	0.1531 (4)	0.7209 (3)	0.0467 (19)	
H80	0.2083	0.1635	0.6841	0.056*	
C81	0.1322 (5)	0.1169 (4)	0.7471 (3)	0.050 (2)	
H81	0.0631	0.1028	0.7284	0.060*	
C82	0.1489 (5)	0.1019 (4)	0.8005 (3)	0.0408 (17)	
H82	0.0911	0.0763	0.8180	0.049*	
C83	0.2473 (5)	0.1235 (3)	0.8282 (3)	0.0330 (15)	
H83	0.2569	0.1142	0.8654	0.040*	
C84	0.3332 (5)	0.1588 (3)	0.8031 (3)	0.0308 (15)	
H84	0.4017	0.1728	0.8225	0.037*	
C85	0.4756 (4)	0.3134 (3)	0.7723 (3)	0.0283 (14)	
C86	0.4497 (5)	0.3222 (4)	0.8289 (3)	0.0423 (17)	
H86	0.3994	0.2850	0.8407	0.051*	
C87	0.4954 (5)	0.3840 (4)	0.8688 (3)	0.0433 (18)	
H87	0.4781	0.3881	0.9082	0.052*	
C88	0.5638 (5)	0.4386 (4)	0.8536 (3)	0.0365 (16)	
H88	0.5976	0.4798	0.8825	0.044*	
C89	0.5857 (6)	0.4353 (4)	0.7958 (4)	0.060 (2)	
H89	0.6314	0.4749	0.7845	0.071*	
C90	0.5389 (5)	0.3720 (4)	0.7542 (3)	0.0488 (19)	
H90	0.5502	0.3693	0.7141	0.059*	
C91	0.5346 (4)	0.1905 (3)	0.7113 (3)	0.0280 (14)	
C92	0.6099 (4)	0.1995 (3)	0.6537 (3)	0.0278 (14)	
C93	0.5898 (5)	0.1915 (4)	0.5319 (3)	0.0356 (16)	
C94	0.5128 (5)	0.1292 (4)	0.5086 (3)	0.0414 (17)	
H94	0.4486	0.1180	0.5269	0.050*	
C95	0.5274 (6)	0.0827 (4)	0.4591 (3)	0.055 (2)	
H95	0.4747	0.0393	0.4442	0.066*	
C96	0.6198 (7)	0.1002 (5)	0.4316 (4)	0.069 (3)	
H96	0.6306	0.0688	0.3977	0.083*	
C97	0.6961 (7)	0.1629 (5)	0.4534 (4)	0.068 (3)	
H97	0.7582	0.1752	0.4336	0.081*	
C98	0.6830 (6)	0.2086 (4)	0.5044 (3)	0.051 (2)	
H98	0.7371	0.2510	0.5202	0.061*	
C99	0.6672 (5)	0.3324 (3)	0.6038 (3)	0.0340 (16)	
C100	0.7553 (5)	0.3659 (4)	0.6419 (3)	0.051 (2)	
H100	0.7718	0.3444	0.6716	0.061*	
C101	0.8203 (6)	0.4314 (4)	0.6366 (4)	0.064 (2)	
H101	0.8814	0.4539	0.6627	0.077*	
C102	0.7983 (6)	0.4630 (4)	0.5956 (4)	0.062 (2)	
H102	0.8439	0.5074	0.5926	0.074*	
C103	0.7088 (6)	0.4314 (4)	0.5573 (3)	0.053 (2)	
H103	0.6927	0.4539	0.5282	0.063*	
C104	0.6433 (5)	0.3663 (4)	0.5624 (3)	0.0442 (18)	
H104	0.5811	0.3448	0.5370	0.053*	
O1	0.5000	0.5000	0.5000	0.124 (4)*	
O2	0.4605 (13)	0.6157 (9)	0.5794 (7)	0.127 (6)*	0.50
C105	0.1611 (7)	0.6054 (5)	0.5840 (4)	0.080 (3)	
H10A	0.1858	0.5627	0.5721	0.096*	
H10B	0.1228	0.6108	0.5496	0.096*	
P1	0.08514 (10)	0.75599 (7)	−0.00144 (6)	0.0181 (3)	
P2	−0.03011 (11)	0.67667 (8)	−0.13385 (6)	0.0206 (3)	
P3	0.20151 (11)	0.85853 (8)	−0.14488 (7)	0.0217 (3)	
P4	0.31718 (11)	0.74770 (8)	−0.09602 (6)	0.0202 (3)	
P5	0.30666 (12)	0.35056 (9)	0.64326 (7)	0.0291 (4)	
P6	0.25284 (12)	0.20160 (9)	0.54478 (7)	0.0267 (4)	
P7	0.56905 (12)	0.24710 (9)	0.60042 (7)	0.0291 (4)	
P8	0.42192 (12)	0.23123 (9)	0.71552 (7)	0.0279 (4)	
Cl1	0.0742 (2)	0.59305 (16)	0.63828 (13)	0.1050 (9)	
Cl2	0.2658 (3)	0.6759 (2)	0.60485 (17)	0.1588 (16)	
Au1	0.138379 (16)	0.761176 (11)	−0.099659 (9)	0.01778 (6)	
Au2	0.393473 (17)	0.255209 (12)	0.619681 (10)	0.02597 (7)	
B1	−0.1165 (5)	0.6728 (4)	0.0408 (3)	0.0311 (17)	
B2	−0.2339 (6)	0.6018 (4)	0.0022 (3)	0.0350 (19)	
B3	−0.2204 (5)	0.6106 (4)	−0.0732 (4)	0.0339 (18)	
B4	−0.2393 (5)	0.6920 (4)	−0.0792 (3)	0.0297 (17)	
H4A	−0.2784	0.6991	−0.1191	0.036*	
B5	−0.1282 (5)	0.7600 (4)	−0.0392 (3)	0.0240 (15)	
H5A	−0.0922	0.8103	−0.0535	0.029*	
B6	−0.1378 (5)	0.7569 (4)	0.0350 (3)	0.0272 (16)	
H6A	−0.1114	0.8063	0.0701	0.033*	
B7	−0.2450 (5)	0.6850 (4)	0.0418 (3)	0.0352 (18)	
H7	−0.2877	0.6870	0.0825	0.042*	
B8	−0.3084 (5)	0.6458 (4)	−0.0307 (3)	0.0352 (18)	
H8A	−0.3959	0.6214	−0.0387	0.042*	
B9	−0.2530 (5)	0.7390 (4)	−0.0096 (3)	0.0308 (17)	
H9A	−0.3033	0.7763	−0.0034	0.037*	
B10	0.5293 (6)	0.8352 (4)	−0.1385 (4)	0.0391 (19)	
H10C	0.5824	0.8252	−0.1058	0.047*	
B11	0.3682 (6)	0.8648 (4)	−0.2384 (3)	0.038 (2)	
H11A	0.3155	0.8744	−0.2716	0.046*	
B12	0.4481 (5)	0.8902 (4)	−0.1182 (4)	0.0339 (18)	
H12A	0.4470	0.9162	−0.0714	0.041*	
B13	0.5539 (6)	0.9185 (4)	−0.1598 (4)	0.044 (2)	
H13	0.6239	0.9644	−0.1410	0.053*	
B14	0.5660 (6)	0.8421 (4)	−0.2099 (4)	0.043 (2)	
H14	0.6445	0.8372	−0.2244	0.052*	
B15	0.4638 (6)	0.7674 (4)	−0.1998 (3)	0.0346 (18)	
H15	0.4739	0.7128	−0.2074	0.041*	
B16	0.3401 (6)	0.7822 (4)	−0.2161 (3)	0.0305 (17)	
H16A	0.2675	0.7376	−0.2333	0.037*	
B17	0.4483 (6)	0.8078 (4)	−0.2583 (4)	0.042 (2)	
H17A	0.4483	0.7795	−0.3046	0.050*	
B18	0.5058 (6)	0.9014 (4)	−0.2333 (4)	0.047 (2)	
H18A	0.5451	0.9355	−0.2631	0.056*	
B19	0.4304 (6)	0.9323 (4)	−0.1773 (3)	0.0381 (19)	
H19A	0.4173	0.9860	−0.1696	0.046*	
B20	0.5211 (6)	0.1188 (4)	0.6543 (3)	0.0318 (17)	
H20A	0.4484	0.0948	0.6237	0.038*	
B21	0.5248 (6)	0.1101 (4)	0.7271 (3)	0.0312 (17)	
H21	0.4539	0.0781	0.7445	0.037*	
B22	0.6095 (5)	0.1903 (4)	0.7701 (3)	0.0303 (17)	
H22A	0.5947	0.2121	0.8160	0.036*	
B23	0.6606 (6)	0.2467 (5)	0.7241 (4)	0.048 (2)	
H23A	0.6789	0.3056	0.7392	0.058*	
B24	0.6517 (6)	0.1244 (4)	0.6306 (3)	0.0331 (17)	
H24A	0.6652	0.1027	0.5844	0.040*	
B25	0.7375 (6)	0.2040 (4)	0.6728 (3)	0.0345 (18)	
H25A	0.8075	0.2351	0.6540	0.041*	
B26	0.7395 (6)	0.1977 (4)	0.7470 (3)	0.0335 (18)	
H26A	0.8121	0.2242	0.7773	0.040*	
B27	0.6529 (6)	0.1135 (4)	0.7508 (3)	0.0350 (18)	
H27	0.6678	0.0845	0.7843	0.042*	
B28	0.5990 (6)	0.0686 (4)	0.6783 (3)	0.0385 (19)	
H28A	0.5781	0.0096	0.6635	0.046*	
B29	0.7325 (6)	0.1222 (4)	0.6904 (3)	0.0383 (19)	
H29A	0.8003	0.0986	0.6841	0.046*	
B30	0.2719 (6)	0.3414 (4)	0.5161 (3)	0.0400 (19)	
H30A	0.3580	0.3485	0.5107	0.048*	
B31	0.1146 (6)	0.3758 (4)	0.5894 (4)	0.0381 (19)	
B32	0.1711 (7)	0.2807 (4)	0.4635 (4)	0.044 (2)	
H32A	0.1914	0.2494	0.4223	0.053*	
B33	0.2236 (7)	0.4129 (4)	0.5491 (4)	0.043 (2)	
H33	0.2776	0.4679	0.5639	0.051*	
B34	0.0908 (7)	0.3956 (5)	0.5209 (4)	0.051 (2)	
H34A	0.0569	0.4400	0.5175	0.062*	
B35	0.0073 (7)	0.3171 (5)	0.5363 (4)	0.052 (2)	
B36	0.0642 (6)	0.2451 (5)	0.5024 (4)	0.041 (2)	
B37	0.0608 (7)	0.3133 (5)	0.4680 (4)	0.048 (2)	
H37A	0.0059	0.3027	0.4284	0.058*	
B38	0.1902 (7)	0.3735 (5)	0.4750 (4)	0.049 (2)	
H38A	0.2214	0.4030	0.4407	0.059*	
H1	−0.067 (5)	0.668 (3)	0.083 (3)	0.059*	
H2A	−0.270 (5)	0.554 (4)	0.017 (3)	0.059*	
H3A	−0.237 (5)	0.562 (4)	−0.119 (3)	0.059*	
H3B	−0.146 (3)	0.618 (2)	0.003 (2)	0.059*	
H31A	0.104 (5)	0.410 (4)	0.631 (3)	0.059*	
H31B	0.064 (4)	0.314 (2)	0.574 (2)	0.059*	
H35A	−0.079 (3)	0.311 (3)	0.539 (3)	0.059*	
H36A	0.022 (5)	0.186 (4)	0.486 (3)	0.059*	

### Atomic displacement parameters (Å^2^)

**Table:**

	*U*^11^	*U*^22^	*U*^33^	*U*^12^	*U*^13^	*U*^23^
C1	0.009 (3)	0.020 (3)	0.025 (3)	0.002 (2)	−0.004 (2)	0.002 (3)
C2	0.029 (3)	0.024 (3)	0.025 (3)	0.009 (3)	−0.001 (3)	0.005 (3)
C3	0.031 (3)	0.025 (3)	0.040 (4)	0.010 (3)	−0.001 (3)	0.003 (3)
C4	0.032 (3)	0.033 (4)	0.035 (4)	0.013 (3)	−0.004 (3)	−0.001 (3)
C5	0.022 (3)	0.056 (5)	0.021 (3)	0.015 (3)	0.003 (3)	0.005 (3)
C6	0.022 (3)	0.032 (4)	0.029 (4)	0.005 (3)	0.007 (3)	0.008 (3)
C7	0.021 (3)	0.022 (3)	0.024 (3)	0.004 (2)	0.003 (2)	0.009 (3)
C8	0.020 (3)	0.022 (3)	0.040 (4)	0.001 (3)	−0.004 (3)	0.010 (3)
C9	0.029 (3)	0.033 (4)	0.043 (4)	0.006 (3)	−0.013 (3)	0.013 (3)
C10	0.038 (4)	0.029 (4)	0.054 (5)	0.012 (3)	−0.002 (3)	0.022 (3)
C11	0.048 (4)	0.023 (4)	0.048 (4)	0.013 (3)	−0.015 (3)	0.006 (3)
C12	0.034 (3)	0.033 (4)	0.028 (4)	0.012 (3)	−0.007 (3)	0.002 (3)
C13	0.017 (3)	0.019 (3)	0.020 (3)	0.006 (2)	0.003 (2)	0.002 (2)
C14	0.014 (3)	0.020 (3)	0.030 (3)	0.005 (2)	−0.003 (2)	0.005 (3)
C15	0.022 (3)	0.032 (4)	0.024 (3)	0.006 (3)	−0.008 (3)	−0.004 (3)
C16	0.028 (3)	0.026 (4)	0.035 (4)	0.005 (3)	−0.001 (3)	0.003 (3)
C17	0.052 (4)	0.028 (4)	0.058 (5)	0.010 (3)	−0.004 (4)	0.007 (4)
C18	0.058 (5)	0.030 (4)	0.050 (5)	0.021 (4)	−0.012 (4)	−0.008 (4)
C19	0.045 (4)	0.046 (5)	0.035 (4)	0.018 (4)	−0.002 (3)	−0.004 (4)
C20	0.033 (3)	0.034 (4)	0.027 (4)	0.012 (3)	0.000 (3)	0.001 (3)
C21	0.016 (3)	0.042 (4)	0.027 (4)	0.007 (3)	0.000 (3)	0.007 (3)
C22	0.028 (4)	0.057 (5)	0.042 (4)	0.021 (3)	0.007 (3)	0.016 (4)
C23	0.051 (5)	0.086 (6)	0.055 (5)	0.042 (5)	0.002 (4)	0.035 (5)
C24	0.066 (6)	0.144 (10)	0.057 (6)	0.064 (7)	−0.009 (5)	0.034 (7)
C25	0.066 (6)	0.114 (9)	0.058 (6)	0.044 (6)	−0.033 (5)	0.000 (6)
C26	0.039 (4)	0.056 (5)	0.045 (5)	0.017 (4)	−0.018 (3)	−0.007 (4)
C27	0.030 (3)	0.019 (3)	0.021 (3)	0.007 (3)	−0.002 (3)	0.000 (3)
C28	0.025 (3)	0.022 (3)	0.051 (4)	0.008 (3)	−0.007 (3)	0.003 (3)
C29	0.042 (4)	0.027 (4)	0.078 (6)	0.011 (3)	−0.019 (4)	0.004 (4)
C30	0.058 (5)	0.033 (4)	0.051 (5)	0.025 (4)	−0.023 (4)	−0.002 (3)
C31	0.038 (4)	0.043 (4)	0.041 (4)	0.022 (3)	−0.009 (3)	−0.006 (3)
C32	0.029 (3)	0.030 (4)	0.028 (4)	0.015 (3)	−0.007 (3)	−0.006 (3)
C33	0.018 (3)	0.020 (3)	0.026 (3)	0.005 (2)	0.005 (2)	0.008 (3)
C34	0.025 (3)	0.027 (3)	0.028 (4)	0.009 (3)	0.006 (3)	0.001 (3)
C35	0.030 (3)	0.034 (4)	0.028 (4)	0.011 (3)	−0.001 (3)	0.010 (3)
C36	0.023 (3)	0.034 (4)	0.024 (3)	−0.001 (3)	−0.001 (3)	0.002 (3)
C37	0.028 (3)	0.023 (3)	0.034 (4)	0.003 (3)	−0.004 (3)	−0.007 (3)
C38	0.022 (3)	0.027 (3)	0.031 (4)	0.006 (3)	0.005 (3)	0.003 (3)
C39	0.019 (3)	0.025 (3)	0.028 (3)	0.005 (3)	0.006 (3)	0.004 (3)
C40	0.024 (3)	0.028 (3)	0.029 (3)	0.010 (3)	0.010 (3)	0.012 (3)
C41	0.026 (3)	0.014 (3)	0.024 (3)	0.008 (2)	0.007 (3)	0.011 (3)
C42	0.033 (3)	0.027 (4)	0.041 (4)	0.014 (3)	0.010 (3)	0.012 (3)
C43	0.041 (4)	0.046 (4)	0.052 (5)	0.027 (4)	0.018 (4)	0.023 (4)
C44	0.059 (5)	0.024 (4)	0.042 (4)	0.020 (3)	0.023 (4)	0.012 (3)
C45	0.042 (4)	0.025 (4)	0.038 (4)	0.001 (3)	0.005 (3)	0.004 (3)
C46	0.025 (3)	0.022 (3)	0.043 (4)	0.010 (3)	0.013 (3)	0.013 (3)
C47	0.026 (3)	0.042 (4)	0.023 (3)	0.010 (3)	0.006 (3)	0.016 (3)
C48	0.062 (5)	0.041 (4)	0.035 (4)	0.015 (4)	−0.002 (4)	0.014 (3)
C49	0.091 (6)	0.063 (6)	0.042 (5)	0.028 (5)	−0.004 (4)	0.026 (5)
C50	0.095 (7)	0.076 (6)	0.032 (5)	0.025 (5)	−0.010 (4)	0.021 (5)
C51	0.051 (5)	0.058 (5)	0.032 (4)	0.002 (4)	−0.004 (3)	0.003 (4)
C52	0.040 (4)	0.035 (4)	0.028 (4)	0.010 (3)	0.006 (3)	0.010 (3)
C58A	0.047 (3)	0.023 (3)	0.046 (3)	−0.003 (2)	−0.003 (2)	0.006 (2)
C53A	0.047 (3)	0.023 (3)	0.046 (3)	−0.003 (2)	−0.003 (2)	0.006 (2)
C55A	0.047 (3)	0.023 (3)	0.046 (3)	−0.003 (2)	−0.003 (2)	0.006 (2)
C56A	0.047 (3)	0.023 (3)	0.046 (3)	−0.003 (2)	−0.003 (2)	0.006 (2)
C57A	0.047 (3)	0.023 (3)	0.046 (3)	−0.003 (2)	−0.003 (2)	0.006 (2)
C54A	0.047 (3)	0.023 (3)	0.046 (3)	−0.003 (2)	−0.003 (2)	0.006 (2)
C53B	0.042 (6)	0.054 (7)	0.111 (9)	−0.001 (5)	0.017 (6)	−0.035 (6)
C58B	0.042 (6)	0.054 (7)	0.111 (9)	−0.001 (5)	0.017 (6)	−0.035 (6)
C55B	0.042 (6)	0.054 (7)	0.111 (9)	−0.001 (5)	0.017 (6)	−0.035 (6)
C54B	0.042 (6)	0.054 (7)	0.111 (9)	−0.001 (5)	0.017 (6)	−0.035 (6)
C56B	0.042 (6)	0.054 (7)	0.111 (9)	−0.001 (5)	0.017 (6)	−0.035 (6)
C57B	0.042 (6)	0.054 (7)	0.111 (9)	−0.001 (5)	0.017 (6)	−0.035 (6)
C59	0.027 (3)	0.023 (3)	0.030 (4)	0.006 (3)	0.003 (3)	−0.004 (3)
C60	0.050 (4)	0.051 (5)	0.042 (5)	0.015 (4)	0.003 (4)	0.007 (4)
C61	0.049 (5)	0.060 (5)	0.061 (6)	0.005 (4)	0.016 (4)	0.018 (5)
C62	0.048 (4)	0.041 (4)	0.041 (5)	0.009 (4)	0.018 (4)	0.015 (4)
C63	0.052 (4)	0.055 (5)	0.023 (4)	0.015 (4)	0.000 (3)	0.007 (3)
C64	0.037 (4)	0.037 (4)	0.032 (4)	0.003 (3)	−0.007 (3)	0.004 (3)
C65	0.032 (3)	0.025 (3)	0.035 (4)	0.006 (3)	0.000 (3)	0.004 (3)
C66	0.030 (3)	0.034 (4)	0.018 (3)	0.002 (3)	−0.007 (3)	−0.004 (3)
C67	0.040 (4)	0.034 (4)	0.018 (3)	0.011 (3)	0.003 (3)	0.005 (3)
C68	0.044 (4)	0.047 (4)	0.041 (4)	0.012 (4)	0.017 (3)	0.017 (4)
C69	0.077 (6)	0.080 (6)	0.054 (6)	0.044 (5)	0.040 (5)	0.036 (5)
C70	0.112 (8)	0.094 (7)	0.027 (5)	0.054 (7)	0.030 (5)	0.010 (5)
C71	0.073 (6)	0.057 (5)	0.035 (5)	0.016 (4)	0.012 (4)	−0.011 (4)
C72	0.054 (4)	0.046 (5)	0.029 (4)	0.012 (4)	0.011 (3)	0.005 (3)
C73	0.027 (3)	0.038 (4)	0.019 (3)	−0.001 (3)	−0.006 (3)	0.006 (3)
C74	0.035 (4)	0.047 (4)	0.025 (4)	0.003 (3)	−0.001 (3)	0.005 (3)
C75	0.037 (4)	0.067 (5)	0.039 (4)	−0.002 (4)	0.003 (3)	0.025 (4)
C76	0.037 (4)	0.069 (6)	0.062 (5)	−0.010 (4)	−0.008 (4)	0.039 (5)
C77	0.054 (5)	0.054 (5)	0.083 (6)	0.007 (4)	−0.001 (4)	0.049 (5)
C78	0.035 (4)	0.046 (4)	0.059 (5)	0.011 (3)	0.002 (3)	0.028 (4)
C79	0.025 (3)	0.034 (4)	0.029 (4)	0.005 (3)	0.005 (3)	0.009 (3)
C80	0.043 (4)	0.054 (5)	0.037 (4)	−0.002 (4)	0.000 (3)	0.018 (4)
C81	0.037 (4)	0.056 (5)	0.045 (5)	−0.006 (4)	−0.002 (3)	0.009 (4)
C82	0.033 (4)	0.046 (4)	0.039 (4)	−0.002 (3)	0.010 (3)	0.017 (4)
C83	0.035 (4)	0.034 (4)	0.034 (4)	0.014 (3)	0.010 (3)	0.010 (3)
C84	0.028 (3)	0.033 (4)	0.034 (4)	0.009 (3)	−0.003 (3)	0.011 (3)
C85	0.025 (3)	0.032 (4)	0.025 (4)	0.004 (3)	0.002 (3)	0.006 (3)
C86	0.046 (4)	0.032 (4)	0.046 (5)	0.007 (3)	0.013 (4)	0.006 (3)
C87	0.047 (4)	0.035 (4)	0.045 (5)	0.013 (4)	0.009 (4)	0.000 (4)
C88	0.029 (3)	0.034 (4)	0.044 (4)	0.013 (3)	−0.010 (3)	−0.001 (3)
C89	0.038 (4)	0.043 (5)	0.083 (7)	−0.006 (4)	0.007 (4)	0.005 (5)
C90	0.040 (4)	0.048 (5)	0.053 (5)	0.002 (4)	0.006 (4)	0.012 (4)
C91	0.022 (3)	0.033 (4)	0.029 (4)	0.008 (3)	0.002 (3)	0.008 (3)
C92	0.021 (3)	0.029 (3)	0.032 (4)	0.005 (3)	0.005 (3)	0.007 (3)
C93	0.034 (4)	0.040 (4)	0.038 (4)	0.013 (3)	0.008 (3)	0.017 (3)
C94	0.046 (4)	0.052 (5)	0.025 (4)	0.012 (4)	0.007 (3)	0.007 (3)
C95	0.057 (5)	0.056 (5)	0.040 (5)	0.005 (4)	0.000 (4)	−0.002 (4)
C96	0.090 (7)	0.062 (6)	0.047 (5)	0.026 (5)	0.022 (5)	−0.010 (4)
C97	0.059 (5)	0.081 (7)	0.057 (6)	0.018 (5)	0.037 (4)	0.004 (5)
C98	0.055 (5)	0.053 (5)	0.042 (5)	0.015 (4)	0.016 (4)	0.003 (4)
C99	0.032 (3)	0.030 (4)	0.037 (4)	0.004 (3)	0.002 (3)	0.006 (3)
C100	0.044 (4)	0.045 (5)	0.056 (5)	−0.001 (4)	−0.014 (4)	0.015 (4)
C101	0.040 (4)	0.047 (5)	0.098 (7)	−0.008 (4)	−0.022 (4)	0.027 (5)
C102	0.044 (4)	0.045 (5)	0.096 (7)	−0.003 (4)	−0.008 (5)	0.035 (5)
C103	0.048 (4)	0.050 (5)	0.062 (5)	0.006 (4)	0.000 (4)	0.029 (4)
C104	0.033 (4)	0.036 (4)	0.058 (5)	−0.006 (3)	−0.011 (3)	0.021 (4)
C105	0.083 (7)	0.068 (6)	0.078 (7)	0.010 (5)	0.010 (5)	0.009 (5)
P1	0.0160 (7)	0.0188 (8)	0.0196 (8)	0.0043 (6)	0.0006 (6)	0.0056 (6)
P2	0.0175 (7)	0.0210 (8)	0.0211 (8)	0.0034 (6)	−0.0016 (6)	0.0028 (7)
P3	0.0224 (8)	0.0203 (8)	0.0248 (8)	0.0080 (6)	0.0050 (6)	0.0074 (7)
P4	0.0168 (7)	0.0182 (8)	0.0247 (8)	0.0053 (6)	0.0015 (6)	0.0022 (7)
P5	0.0263 (8)	0.0286 (9)	0.0272 (9)	0.0044 (7)	0.0012 (7)	−0.0009 (7)
P6	0.0246 (8)	0.0302 (9)	0.0210 (9)	0.0026 (7)	0.0011 (7)	0.0031 (7)
P7	0.0259 (8)	0.0335 (10)	0.0265 (9)	0.0055 (7)	0.0014 (7)	0.0077 (8)
P8	0.0250 (8)	0.0331 (9)	0.0232 (9)	0.0029 (7)	0.0015 (7)	0.0080 (7)
Cl1	0.130 (2)	0.102 (2)	0.093 (2)	0.0300 (19)	0.0296 (18)	0.0456 (17)
Cl2	0.137 (3)	0.148 (3)	0.142 (3)	−0.047 (3)	−0.008 (2)	0.040 (3)
Au1	0.01483 (11)	0.01812 (12)	0.02088 (13)	0.00437 (9)	0.00106 (9)	0.00583 (10)
Au2	0.02331 (13)	0.02883 (15)	0.02257 (14)	0.00246 (11)	0.00025 (10)	0.00535 (11)
B1	0.022 (4)	0.037 (4)	0.034 (4)	0.003 (3)	0.008 (3)	0.012 (4)
B2	0.022 (4)	0.037 (5)	0.043 (5)	−0.003 (3)	0.010 (3)	0.019 (4)
B3	0.023 (4)	0.025 (4)	0.046 (5)	−0.003 (3)	0.002 (3)	0.003 (4)
B4	0.013 (3)	0.034 (4)	0.039 (4)	0.004 (3)	0.000 (3)	0.003 (4)
B5	0.015 (3)	0.023 (4)	0.035 (4)	0.010 (3)	0.002 (3)	0.003 (3)
B6	0.020 (3)	0.034 (4)	0.027 (4)	0.011 (3)	0.005 (3)	0.002 (3)
B7	0.023 (4)	0.040 (5)	0.040 (5)	0.001 (3)	0.006 (3)	0.016 (4)
B8	0.015 (3)	0.042 (5)	0.042 (5)	−0.002 (3)	0.003 (3)	0.007 (4)
B9	0.019 (3)	0.037 (4)	0.034 (4)	0.008 (3)	0.001 (3)	0.003 (4)
B10	0.025 (4)	0.033 (4)	0.058 (5)	0.009 (3)	0.011 (4)	0.006 (4)
B11	0.057 (5)	0.036 (5)	0.033 (5)	0.021 (4)	0.026 (4)	0.019 (4)
B12	0.028 (4)	0.024 (4)	0.046 (5)	0.005 (3)	0.004 (4)	0.004 (4)
B13	0.028 (4)	0.036 (5)	0.064 (6)	0.004 (4)	0.023 (4)	0.009 (4)
B14	0.038 (4)	0.042 (5)	0.054 (5)	0.013 (4)	0.027 (4)	0.016 (4)
B15	0.034 (4)	0.032 (4)	0.032 (4)	0.004 (3)	0.018 (3)	0.001 (4)
B16	0.037 (4)	0.029 (4)	0.023 (4)	0.007 (3)	0.005 (3)	0.002 (3)
B17	0.052 (5)	0.041 (5)	0.036 (5)	0.017 (4)	0.027 (4)	0.011 (4)
B18	0.050 (5)	0.039 (5)	0.055 (6)	0.010 (4)	0.033 (4)	0.018 (4)
B19	0.043 (4)	0.029 (4)	0.045 (5)	0.004 (4)	0.019 (4)	0.018 (4)
B20	0.034 (4)	0.028 (4)	0.027 (4)	−0.003 (3)	−0.005 (3)	0.007 (3)
B21	0.033 (4)	0.027 (4)	0.031 (4)	0.002 (3)	−0.003 (3)	0.011 (3)
B22	0.032 (4)	0.036 (4)	0.024 (4)	0.009 (3)	−0.002 (3)	0.011 (3)
B23	0.041 (5)	0.045 (5)	0.055 (6)	0.007 (4)	0.002 (4)	0.011 (5)
B24	0.036 (4)	0.035 (4)	0.032 (4)	0.018 (4)	0.006 (3)	0.005 (4)
B25	0.027 (4)	0.035 (4)	0.042 (5)	0.011 (3)	0.005 (3)	0.006 (4)
B26	0.025 (4)	0.036 (4)	0.038 (5)	0.006 (3)	−0.001 (3)	0.008 (4)
B27	0.039 (4)	0.036 (5)	0.034 (4)	0.015 (4)	0.000 (4)	0.010 (4)
B28	0.048 (5)	0.027 (4)	0.039 (5)	0.010 (4)	−0.002 (4)	0.005 (4)
B29	0.035 (4)	0.047 (5)	0.040 (5)	0.022 (4)	0.002 (4)	0.011 (4)
B30	0.042 (5)	0.040 (5)	0.037 (5)	0.008 (4)	0.000 (4)	0.011 (4)
B31	0.036 (4)	0.040 (5)	0.037 (5)	0.017 (4)	−0.006 (4)	−0.002 (4)
B32	0.062 (5)	0.046 (5)	0.030 (5)	0.022 (4)	−0.003 (4)	0.013 (4)
B33	0.050 (5)	0.039 (5)	0.041 (5)	0.014 (4)	−0.005 (4)	0.010 (4)
B34	0.067 (6)	0.056 (6)	0.043 (5)	0.036 (5)	−0.014 (4)	0.012 (4)
B35	0.048 (5)	0.055 (6)	0.052 (6)	0.024 (5)	−0.014 (4)	−0.002 (5)
B36	0.043 (5)	0.043 (5)	0.035 (5)	0.012 (4)	−0.014 (4)	0.002 (4)
B37	0.055 (5)	0.053 (6)	0.033 (5)	0.022 (4)	−0.024 (4)	−0.004 (4)
B38	0.076 (6)	0.051 (6)	0.033 (5)	0.030 (5)	0.005 (4)	0.018 (4)

### Geometric parameters (Å)

**Table:**

C1—C2	1.382 (7)	C80—C81	1.405 (9)
C1—C6	1.404 (8)	C80—H80	0.9500
C1—P1	1.827 (6)	C81—C82	1.382 (9)
C2—C3	1.390 (8)	C81—H81	0.9500
C2—H2	0.9500	C82—C83	1.360 (8)
C3—C4	1.374 (9)	C82—H82	0.9500
C3—H3	0.9500	C83—C84	1.378 (8)
C4—C5	1.373 (9)	C83—H83	0.9500
C4—H4	0.9500	C84—H84	0.9500
C5—C6	1.376 (8)	C85—C86	1.370 (9)
C5—H5	0.9500	C85—C90	1.400 (9)
C6—H6	0.9500	C85—P8	1.852 (6)
C7—C8	1.382 (7)	C86—C87	1.371 (9)
C7—C12	1.389 (8)	C86—H86	0.9500
C7—P1	1.833 (6)	C87—C88	1.336 (9)
C8—C9	1.388 (8)	C87—H87	0.9500
C8—H8	0.9500	C88—C89	1.390 (10)
C9—C10	1.380 (9)	C88—H88	0.9500
C9—H9	0.9500	C89—C90	1.413 (10)
C10—C11	1.397 (8)	C89—H89	0.9500
C10—H10	0.9500	C90—H90	0.9500
C11—C12	1.374 (8)	C91—B22	1.693 (9)
C11—H11	0.9500	C91—C92	1.706 (8)
C12—H12	0.9500	C91—B21	1.710 (9)
C13—C14	1.625 (8)	C91—B23	1.719 (10)
C13—B1	1.645 (8)	C91—B20	1.722 (9)
C13—B5	1.719 (8)	C91—P8	1.879 (6)
C13—B6	1.737 (8)	C92—B25	1.711 (9)
C13—P1	1.839 (5)	C92—B20	1.729 (9)
C14—B3	1.621 (8)	C92—B24	1.735 (9)
C14—B4	1.756 (8)	C92—B23	1.751 (11)
C14—B5	1.762 (9)	C92—P7	1.884 (6)
C14—P2	1.851 (6)	C93—C94	1.380 (9)
C15—C20	1.394 (8)	C93—C98	1.392 (9)
C15—C16	1.403 (8)	C93—P7	1.834 (7)
C15—P2	1.830 (6)	C94—C95	1.388 (10)
C16—C17	1.388 (9)	C94—H94	0.9500
C16—H16	0.9500	C95—C96	1.384 (10)
C17—C18	1.365 (10)	C95—H95	0.9500
C17—H17	0.9500	C96—C97	1.375 (11)
C18—C19	1.387 (10)	C96—H96	0.9500
C18—H18	0.9500	C97—C98	1.397 (10)
C19—C20	1.376 (9)	C97—H97	0.9500
C19—H19	0.9500	C98—H98	0.9500
C20—H20	0.9500	C99—C100	1.378 (9)
C21—C22	1.381 (9)	C99—C104	1.383 (9)
C21—C26	1.389 (9)	C99—P7	1.833 (6)
C21—P2	1.827 (6)	C100—C101	1.395 (9)
C22—C23	1.374 (9)	C100—H100	0.9500
C22—H22	0.9500	C101—C102	1.332 (10)
C23—C24	1.358 (12)	C101—H101	0.9500
C23—H23	0.9500	C102—C103	1.390 (10)
C24—C25	1.379 (13)	C102—H102	0.9500
C24—H24	0.9500	C103—C104	1.389 (9)
C25—C26	1.403 (10)	C103—H103	0.9500
C25—H25	0.9500	C104—H104	0.9500
C26—H26	0.9500	C105—Cl2	1.669 (9)
C27—C32	1.389 (8)	C105—Cl1	1.744 (9)
C27—C28	1.398 (8)	C105—H10A	0.9900
C27—P4	1.815 (6)	C105—H10B	0.9900
C28—C29	1.387 (9)	P1—Au1	2.4444 (14)
C28—H28	0.9500	P2—Au1	2.4082 (14)
C29—C30	1.386 (9)	P3—Au1	2.3852 (14)
C29—H29	0.9500	P4—Au1	2.4512 (14)
C30—C31	1.392 (9)	P5—Au2	2.4665 (17)
C30—H30	0.9500	P6—Au2	2.3956 (15)
C31—C32	1.374 (8)	P7—Au2	2.4113 (16)
C31—H31	0.9500	P8—Au2	2.4517 (16)
C32—H32	0.9500	B1—B7	1.780 (10)
C33—C34	1.394 (8)	B1—B6	1.823 (10)
C33—C38	1.396 (8)	B1—B2	1.865 (10)
C33—P4	1.833 (6)	B1—H1	1.20 (7)
C34—C35	1.375 (8)	B1—H3B	1.23 (4)
C34—H34	0.9500	B2—B8	1.760 (11)
C35—C36	1.389 (8)	B2—B7	1.784 (11)
C35—H35	0.9500	B2—B3	1.830 (11)
C36—C37	1.362 (8)	B2—H2A	1.08 (7)
C36—H36	0.9500	B2—H3B	1.11 (4)
C37—C38	1.384 (8)	B3—B8	1.744 (10)
C37—H37	0.9500	B3—B4	1.757 (10)
C38—H38	0.9500	B3—H3A	1.27 (7)
C39—B10	1.710 (9)	B4—B8	1.738 (10)
C39—B12	1.710 (9)	B4—B9	1.753 (10)
C39—C40	1.716 (8)	B4—B5	1.799 (9)
C39—B15	1.721 (9)	B4—H4A	1.1200
C39—B16	1.735 (9)	B5—B6	1.768 (10)
C39—P4	1.875 (6)	B5—B9	1.778 (9)
C40—B19	1.700 (9)	B5—H5A	1.1200
C40—B16	1.722 (9)	B6—B9	1.744 (9)
C40—B11	1.733 (9)	B6—B7	1.764 (9)
C40—B12	1.745 (9)	B6—H6A	1.1200
C40—P3	1.882 (5)	B7—B9	1.809 (10)
C41—C42	1.391 (8)	B7—B8	1.809 (11)
C41—C46	1.395 (8)	B7—H7	1.1200
C41—P3	1.838 (6)	B8—B9	1.776 (10)
C42—C43	1.377 (9)	B8—H8A	1.1200
C42—H42	0.9500	B9—H9A	1.1200
C43—C44	1.376 (9)	B10—B12	1.746 (10)
C43—H43	0.9500	B10—B14	1.781 (11)
C44—C45	1.370 (9)	B10—B15	1.785 (11)
C44—H44	0.9500	B10—B13	1.799 (11)
C45—C46	1.386 (8)	B10—H10C	1.1200
C45—H45	0.9500	B11—B18	1.756 (11)
C46—H46	0.9500	B11—B17	1.763 (11)
C47—C52	1.391 (9)	B11—B19	1.770 (12)
C47—C48	1.398 (9)	B11—B16	1.795 (10)
C47—P3	1.820 (6)	B11—H11A	1.1200
C48—C49	1.381 (9)	B12—B13	1.755 (10)
C48—H48	0.9500	B12—B19	1.817 (11)
C49—C50	1.376 (11)	B12—H12A	1.1200
C49—H49	0.9500	B13—B18	1.765 (12)
C50—C51	1.383 (10)	B13—B14	1.778 (11)
C50—H50	0.9500	B13—B19	1.784 (11)
C51—C52	1.371 (9)	B13—H13	1.1200
C51—H51	0.9500	B14—B18	1.772 (12)
C52—H52	0.9500	B14—B15	1.788 (10)
C58A—C53A	1.3900	B14—B17	1.795 (12)
C58A—C54A	1.3900	B14—H14	1.1200
C58A—H58A	0.9500	B15—B17	1.779 (11)
C53A—C55A	1.3900	B15—B16	1.789 (10)
C53A—P5	1.833 (6)	B15—H15	1.1200
C55A—C56A	1.3900	B16—B17	1.776 (10)
C55A—H55A	0.9500	B16—H16A	1.1200
C56A—C57A	1.3900	B17—B18	1.793 (11)
C56A—H56A	0.9500	B17—H17A	1.1200
C57A—C54A	1.3900	B18—B19	1.776 (11)
C57A—H57A	0.9500	B18—H18A	1.1200
C54A—H54A	0.9500	B19—H19A	1.1200
C53B—C58B	1.3900	B20—B21	1.764 (10)
C53B—C57B	1.3900	B20—B28	1.786 (10)
C53B—P5	1.792 (14)	B20—B24	1.802 (10)
C58B—C55B	1.3900	B20—H20A	1.1200
C58B—H58B	0.9500	B21—B28	1.748 (11)
C55B—C54B	1.3900	B21—B27	1.752 (10)
C55B—H55B	0.9500	B21—B22	1.783 (10)
C54B—C56B	1.3900	B21—H21	1.1200
C54B—H54B	0.9500	B22—B23	1.744 (11)
C56B—C57B	1.3900	B22—B27	1.768 (10)
C56B—H56B	0.9500	B22—B26	1.786 (10)
C57B—H57B	0.9500	B22—H22A	1.1200
C59—C64	1.354 (8)	B23—B26	1.766 (11)
C59—C60	1.393 (9)	B23—B25	1.819 (11)
C59—P5	1.833 (6)	B23—H23A	1.1200
C60—C61	1.403 (10)	B24—B29	1.772 (10)
C60—H60	0.9500	B24—B28	1.773 (10)
C61—C62	1.390 (10)	B24—B25	1.775 (11)
C61—H61	0.9500	B24—H24A	1.1200
C62—C63	1.347 (9)	B25—B29	1.769 (11)
C62—H62	0.9500	B25—B26	1.780 (11)
C63—C64	1.389 (9)	B25—H25A	1.1200
C63—H63	0.9500	B26—B29	1.780 (11)
C64—H64	0.9500	B26—B27	1.788 (10)
C65—C66	1.618 (8)	B26—H26A	1.1200
C65—B31	1.640 (10)	B27—B28	1.790 (11)
C65—B30	1.715 (10)	B27—B29	1.790 (11)
C65—B33	1.735 (10)	B27—H27	1.1200
C65—P5	1.846 (6)	B28—B29	1.781 (11)
C66—B36	1.636 (9)	B28—H28A	1.1200
C66—B32	1.736 (9)	B29—H29A	1.1200
C66—B30	1.752 (10)	B30—B33	1.770 (12)
C66—P6	1.852 (6)	B30—B38	1.770 (11)
C67—C72	1.380 (9)	B30—B32	1.797 (11)
C67—C68	1.393 (8)	B30—H30A	1.1200
C67—P6	1.817 (6)	B31—B34	1.786 (11)
C68—C69	1.378 (10)	B31—B33	1.812 (12)
C68—H68	0.9500	B31—B35	1.841 (12)
C69—C70	1.391 (12)	B31—H31A	1.10 (7)
C69—H69	0.9500	B31—H31B	1.22 (4)
C70—C71	1.353 (11)	B32—B37	1.750 (11)
C70—H70	0.9500	B32—B36	1.765 (12)
C71—C72	1.383 (9)	B32—B38	1.777 (12)
C71—H71	0.9500	B32—H32A	1.1200
C72—H72	0.9500	B33—B38	1.754 (11)
C73—C78	1.401 (9)	B33—B34	1.781 (11)
C73—C74	1.410 (8)	B33—H33	1.1200
C73—P6	1.826 (6)	B34—B35	1.775 (13)
C74—C75	1.409 (9)	B34—B37	1.794 (12)
C74—H74	0.9500	B34—B38	1.803 (13)
C75—C76	1.380 (10)	B34—H34A	1.1200
C75—H75	0.9500	B35—B37	1.762 (13)
C76—C77	1.383 (10)	B35—B36	1.854 (12)
C76—H76	0.9500	B35—H31B	1.18 (4)
C77—C78	1.378 (9)	B35—H35A	1.12 (4)
C77—H77	0.9500	B36—B37	1.746 (12)
C78—H78	0.9500	B36—H36A	1.14 (7)
C79—C80	1.385 (8)	B37—B38	1.787 (13)
C79—C84	1.394 (8)	B37—H37A	1.1200
C79—P8	1.835 (6)	B38—H38A	1.1200
